# Determinants of Spatial Distribution in a Bee Community: Nesting Resources, Flower Resources, and Body Size

**DOI:** 10.1371/journal.pone.0097255

**Published:** 2014-05-13

**Authors:** Anna Torné-Noguera, Anselm Rodrigo, Xavier Arnan, Sergio Osorio, Helena Barril-Graells, Léo Correia da Rocha-Filho, Jordi Bosch

**Affiliations:** 1 CREAF, Cerdanyola del Vallès, Catalunya, Spain; 2 Unitat d'Ecologia, Univ Autònoma de Barcelona, Cerdanyola del Vallès, Catalunya, Spain; 3 Fachbereich Biologie, Technische Universität Darmstadt, Darmstadt, Germany; 4 Departamento de Biologia, Universidade de São Paulo, Ribeirão Preto, Brazil; University of Northampton, United Kingdom

## Abstract

Understanding biodiversity distribution is a primary goal of community ecology. At a landscape scale, bee communities are affected by habitat composition, anthropogenic land use, and fragmentation. However, little information is available on local-scale spatial distribution of bee communities within habitats that are uniform at the landscape scale. We studied a bee community along with floral and nesting resources over a 32 km^2^ area of uninterrupted Mediterranean scrubland. Our objectives were (i) to analyze floral and nesting resource composition at the habitat scale. We ask whether these resources follow a geographical pattern across the scrubland at bee-foraging relevant distances; (ii) to analyze the distribution of bee composition across the scrubland. Bees being highly mobile organisms, we ask whether bee composition shows a homogeneous distribution or else varies spatially. If so, we ask whether this variation is irregular or follows a geographical pattern and whether bees respond primarily to flower or to nesting resources; and (iii) to establish whether body size influences the response to local resource availability and ultimately spatial distribution. We obtained 6580 specimens belonging to 98 species. Despite bee mobility and the absence of environmental barriers, our bee community shows a clear geographical pattern. This pattern is mostly attributable to heterogeneous distribution of small (<55 mg) species (with presumed smaller foraging ranges), and is mostly explained by flower resources rather than nesting substrates. Even then, a large proportion (54.8%) of spatial variability remains unexplained by flower or nesting resources. We conclude that bee communities are strongly conditioned by local effects and may exhibit spatial heterogeneity patterns at a scale as low as 500–1000 m in patches of homogeneous habitat. These results have important implications for local pollination dynamics and spatial variation of plant-pollinator networks.

## Introduction

From a strictly theoretical perspective, a community may be defined as the assemblage of species occupying an area within which all individuals are equally likely to interact, thus hindering spatial heterogeneity in distribution or abundance [1]. However, we live in a highly heterogeneous world, and even the most uniform habitats show important levels of spatial variability in environmental conditions at one scale or another. From a more deterministic perspective, species composition is expected to be closely related to this within-habitat heterogeneity, for example in resource availability [2]. However, the effects of resource distribution on community composition may be difficult to predict for several reasons. First, different species may respond to resource distribution at different scales. Large species, with greater food requirements and greater mobility are expected to respond to resource distribution at larger scales [3]. Small species, on the other hand, may be able to satisfy their needs within a small area and therefore be more sensitive to local scale factors. Second, a given species may depend on various resources with differing distribution patterns, and thus respond to each resource at a different scale [4]. Local community structure is further shaped by species' functional traits, such as dispersal ability, and by interactions between species resulting in either avoidance or attraction [5]. Finally, community structure may be historically contingent, so that even under similar environmental conditions, different species assemblages may arise as a result of different immigration history or disturbance events [6].

In this study we analyze the spatial distribution of a bee community as well as the distribution of the nesting and floral resources on which bees depend. Most bee species build nests and provision them with pollen and nectar as food for their larvae. Once a bee has established at a nesting site, it conducts repeated pollen-nectar foraging trips, thus becoming a central place forager. Because different species use different nesting substrates and favour different pollen sources, bee diversity is expected to be higher in areas hosting a variety of nesting and floral resources [7]. Pollen specialization in bees ranges from polylecty (species collecting pollen from many unrelated plant families), to oligolecty (collecting pollen from a single plant family), and monolecty (collecting pollen from a single plant genus). As for nesting substrates, most bee species excavate their nests underground, but some do so in dead wood or in soft-pith stems. Other species exploit different types of pre-existing cavities, and a smaller number build exposed nests attached to rocks or to the vegetation. Finally, some bee species are cleptoparasitic, laying their eggs in nests of other bee species, usually of a given genus. A number of studies have documented the influence of flower resources on the structure of bee communities [8]–[15]. Fewer studies have addressed the role of nesting substrates [9], [13], [16]–[18], and establishing the relative importance of flower *versus* nesting resources has become a key topic in bee ecology research [19]. While attaching a greater weight to flower resources, the review of Roulston and Goodell [7] emphasizes the need to consider both types of resources, partly because of the spatial complexity of resource distribution and partly because nesting substrate diversity is often correlated with plant diversity.

Bees are able to fly long distances and therefore have the capacity to readily colonize suitable sites. Several studies have estimated bee foraging ranges through the use of various techniques, including measures of trip duration, experiments of homing ability, harmonic radar tracking, mark-recapture experiments and genetic analysis of foraging bees [20]–[28]. These studies indicate that most species forage within a few hundred meters from their nest but some may fly thousands of meters. These studies also show a consistent positive relationship between body size and estimated foraging distance. We may thus expect species of different body sizes to respond differently to spatial resource distribution.

Previous studies have shown differences in bee community composition at landscape scales and in relation to habitat composition, anthropogenic land use change and fragmentation [18], [29]–[33]. However, we know of no studies exploring the distribution of an entire bee community at a local scale within a habitat that may be considered homogeneous at a landscape scale. This scale is important because most individual bee movements probably occur at this scale. Our study was conducted in an area covered by contiguous Mediterranean scrubland, with uniform climatic conditions and no ecological or physical barriers. Because bees are highly mobile, one might expect within-habitat differences in bee distribution to be small. However, a few studies have shown that pollinator assemblages visiting various plant-species may vary at scales of hundreds or even tens of meters [34]–[36].

Most models on community assembly dynamics assume that environmental conditions are homogeneous across a patch of uniform habitat, although this assumption is clearly not met in many systems [6]. Our first objective is to analyze floral and nesting resource composition heterogeneity at the habitat scale. We ask whether this heterogeneity is irregular or else follows a geographical pattern across the scrubland at bee-foraging relevant distances. Our second objective is to analyze the distribution of bee composition across the habitat. A homogeneous distribution would be in agreement with the above-mentioned theoretical definition of community [1], and would reflect high levels of connection among plots, either through foraging movements, through dispersal rates, or both. Given the size of the area sampled (5.4 km by 6.2 km) and the high degree of mobility displayed by bees, we assume that any bee species is able to colonize a suitable plot in our study area over one or a few generations. Alternatively, bee composition might show a heterogeneous distribution if bee foraging areas were small and bee distribution closely tracked spatial variation in resource availability at the local scale. If the latter, we ask whether bees respond primarily to flower or to nesting resource distribution. Our bee community is rich (98 species) and encompasses a wide range of body sizes and therefore presumed energetic requirements and mobility. Our third objective is to establish whether species with different body sizes respond differently to local resource availability and show different patterns of spatial distribution.

## Materials and Methods

### Ethics Statement

All necessary permits were obtained for the described study. Field work was conducted with permission of Diputació de Barcelona and the park's administration.

### Study Area

The study was conducted in the Natural Park of Garraf (Barcelona, NE Spain). We selected 21 plots of 40 m×40 m distributed more or less regularly across the park, encompassing an overall area of 32 km^2^. Distances between nearest plots ranged from 585 to 1354 m. The two most distant plots were 6.2 km apart. Plots ranged in altitude from 255 to 545 m, and their distance to the coast ranged from 1500 to 6800 m. At a landscape scale, the study area can be considered homogeneous. The 21 selected plots share the same vegetation type, soil type and recent disturbance history. Physical or environmental barriers are lacking and there are no significant climatic gradients. The park is located on a karstic massif of limestone and dolostone. This soil type favours drainage, thus hindering water storage. Stream beds are lacking and none of the plots is located at the bottom of a valley. The area is occupied by a Mediterranean scrubland. Plant composition varies locally from plot to plot, but is always largely dominated by *Quercus coccifera*, *Pistacia lentiscus*, *Rosmarinus officinalis* and *Thymus vulgaris*.

### Bee Sampling

We conducted 8 surveys (one every two weeks) from mid March to late June 2010, thus encompassing the main flowering period of the scrubland (flowers are very scarce in July and August). To avoid the influence of weather conditions, surveys were conducted simultaneously in all plots. In each survey we placed 6 sampling stations in two parallel rows, with a distance of 10 m between stations. Following Westphal et al. [37], each station was composed of a metal bar holding 3 pan traps (15-cm-diameter plastic bowls painted yellow, white and blue, respectively, with UV-reflecting paint). Traps were located at 20–40 cm above ground level and approximately 50 cm away from the nearest flowering plant. Before 9:30 on each sampling day, traps were filled with water containing a small amount of detergent and collected after 18:00, thus covering most of the daily activity period. Pan trapping has been shown to underestimate bee richness and to provide an incomplete measure of flower visitation compared to netting of flower visiting insects [37], [38]. However, our main concerns were to sample all 21 plots simultaneously, to avoid collector bias, and to apply the same sampling effort to each plot. Our goal was to characterize the bee community, rather than sample bee-flower interactions.

Captured specimens were dried and pinned for identification in the laboratory. From these samples we obtained measures of species richness (number of species captured), abundance (number of individuals captured) and composition (abundance of each species) for each plot. Fresh body weights were obtained from netted specimens. All specimens were weighed a few hours after being captured and, inasmuch as possible, we measured more than one specimen per species (mean = 7.4; range = 1–52). We use female weight in all analyses.

### Flower Resources

To estimate flower richness we counted all flower species along two 40 m×1 m transects arranged as an X centred in the centre of the sampling station grid. This was done three times, in mid April, mid May and mid June. In addition, we estimated flower density of the main flowering species (*R. officinalis*, *T. vulgaris*, *Dorycnium pentaphyllum*, *Cistus albidus*, *Cistus salvifolius* and *Cistus monspeliensis*) in each plot. These species represent 70–90% of the flowers produced in the study area (unpublished data from weekly flower counts in transects at 12 different sites across the park). We first calculated the volume of each flower patch in the transects by measuring two perpendicular widths and the height. Then, to establish a relationship between patch volume and number of flowers, we counted all open flowers in a subsample of patches (n = 59–226 per species) at peak bloom (Linear regression: R^2^ = 0.36–0.63, *P* = 0.001–0.015). The three *Cistus* species were scarce compared to the other species and their blooming periods overlapped widely. Therefore, we lumped together the three species in a single variable (*Cistus* flowers). In an attempt to tease apart the effects of pollen and nectar we used measures of pollen and nectar production per flower of each species (unpublished data) to estimate pollen and nectar density in each plot. However, these two variables were highly correlated (r = 0.96, p<0.0001), and they were also correlated to flower density (r = 0.82, p<0.0001 and r = 0.77, p<0.0001, respectively). Therefore, we use flower density in all analyses.

### Nesting Substrates

We used the above-mentioned transects to measure availability of nesting substrates. On every m^2^ of transect we placed a wire grid delimiting 32 cells (each measuring 0.031 m^2^), and each cell was scored as containing one or no potential nesting substrates. We used the following nesting substrate variables: % bare soil, % bare soil with stones, presence of dead wood, number of holes in rocks, number of vacant snail shells, % *Quercus coccifera* cover, and % *Ampelodesmos mauritanica* cover. *Quercus coccifera* was included because we often observed *Bombus terrestris* bumblebees nesting at their base. *Ampelodesmos mauritanica* was included because it produces soft-pith and hollow stems that might be used by some bee species in the genera *Ceratina, Heriades, Protosmia* and *Hoplitis*.

### Statistical Analysis

All flower resource variables were square-root transformed to improve normality and homoscedasticity. Nesting resource variables were log transformed, except *Q. coccifera* cover, which was square-root transformed. Bare soil and bare soil with stones were significantly correlated (r = 0.67, p = 0.001) and thus we lumped them together in a single variable (bare soil cover). The remaining resource variables were not significantly correlated.

We used Moran's I correlograms to explore spatial distribution of flower richness, flower density of each sampled species, overall flower abundance, cover of each nesting substrate, bee species richness, overall bee abundance, and bee abundance of each of the 19 most abundant species (those representing more than 0.5% of the total individuals captured). For the variable “presence of dead wood” we used the binary Join-Count correlogram. To explore spatial distribution of bee community composition, we used a Mantel's correlogram obtained from a matrix of geographical distances and a matrix of similarity (Sørensen's index) of bee species composition. The number of intervals in all correlograms was calculated based on Sturge's rule. Significance of each correlogram was tested through 300 permutations and p-values were applied a progressive Bonferroni correction. To further explore spatial distribution of bee composition, we run a cluster analysis to group plots according to bee composition similarity using UPGMA linkage rule and Euclidean distances, and represented the resulting groups on a map of the study area. These analyses were conducted with the statistical package Ape in R [39] and the software SAM v.4.0 [40].

The relationship between bee species richness and flower richness, between bee abundance and overall flower abundance, and between bee abundance and bee richness was analyzed with simple linear regression. The contribution of flower (flower density of *R. officinalis*, *T. vulgaris*, *D. pentaphyllum* and *Cistus*) and nesting resource (presence of dead wood, % bare soil, number of holes in rocks and number of vacant snail shells) variables to bee species richness and bee abundance was analyzed with general linear models. *Quercus coccifera* cover and *A. mauritanica* cover were not included in these analyses because our Redundancy Analysis (see below) could not find any species associated to these substrates. We selected the most parsimonious model based on Akaike's Information Criterion (AIC) using the step AIC function with forward and backward elimination implemented in the MASS library [41] of the R software [39]. Since neither bee species richness nor abundance were autocorrelated (see results), we did not include spatial variables in these analyses.

To establish the relationship between the spatial distribution of bee composition and flower and nesting resources we conducted an ordination analysis. We first run a detrended correspondence analysis (DCCA) to determine whether our data had a unimodal or a linear response [42]. The results of this analysis showed that our data were sufficiently homogeneous and conformed to a model with a linear response. We thus applied a Redundancy Analysis (RDA). We used the software Canoco v.4.5 to do these analyses [43]. Because body weight clearly conditioned bee spatial distribution, we run two RDAs, one including only small species (fresh body weight <55 mg) and the other including only large species (>70 mg). In both analysis, species abundance data were square-root transformed and centred. Because we did not want to attach too much weight to rare species (the majority) we did not standardize abundance data. In view of the results obtained in the cluster analysis, geographical coordinates were introduced as covariables. Resource variables were automatically selected with the *forward* option, and significance of each variable and significance of the overall model were tested with Monte Carlo simulations under reduced model (499 permutations).

## Results

### Bee Community

We captured 6580 specimens corresponding to 98 species in five families: Apidae (27 species), Megachilidae (26), Andrenidae (23), Halictidae (18) and Colletidae (4) (Table S1). Nineteen species represented 93.2% of the specimens captured, and 30 of the remaining 79 species were singletons. *Lasioglossum subhirtum* was the most abundant species (27.1% of total specimens), followed by *Andrena djelfensis* (14.1%). Plot species richness ranged between 24 and 44, and abundance between 207 and 559. The relationship between bee species richness and abundance failed significance (r^2^ = 0.15; p = 0.09).

### Spatial Distribution of Flower and Nesting Resources

**Figure 1:**
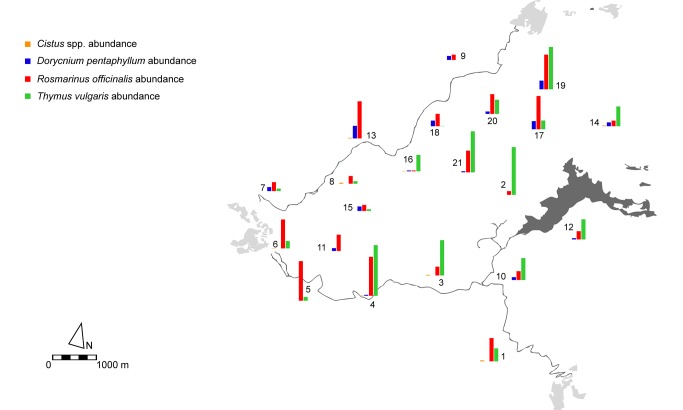
Map of the Garraf Park showing the density of flower resources (number of flowers/m^2^) in each plot (n = 21).

Both flower density (27 to 265 flowers/m^2^) and species richness (5 to 27) varied widely across plots (Table S2 and S3). Flower abundance and richness were not related (r^2^ = 0.07; p>0.25). Flower abundance did not show spatial autocorrelation (I = −0.024, p = 0.51). Instead, flower species richness was significantly autocorrelated (I = 0.186, p<0.0001), with a gradient of positive autocorrelation at short distances (<1000 m) progressively losing significance at longer distances. The only flower species with a significant Moran's I was *T. vulgaris* (I = 0.049, p = 0.015) (Fig. 1). The associated correlogram again showed a gradient of positive autocorrelation at short distance classes with a progressive loss of significance. *Rosmarinus officinalis* was more or less evenly distributed throughout the park, whereas *D. pentaphyllum* was most abundant in the north-western edge. *Cistus* spp. flower density was low compared to the other species, and varied from plot to plot showing no clear pattern (Fig. 1; Table S2).

**Figure 2:**
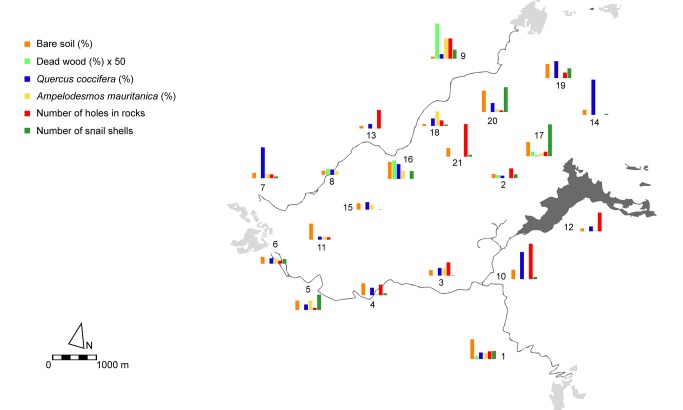
Map of the Garraf Park showing the abundance of nesting resources in each plot (n = 21).

Nesting substrate composition also varied widely across plots (Fig. 2). Bare soil and *Q. coccifera* cover were the only two nesting substrates present in all plots. However, all plots except one offered at least 4 of the 6 nesting resources. The spatial distribution of nesting substrates was highly heterogeneous (Fig. 2, Table S2). None of the nesting substrates showed a discernable spatial pattern, except for holes in rocks (I = 0.045, p = 0.02), again showing decreasing positive autocorrelation with increasing distance.

### Bee Spatial Distribution

**Figure 3:**
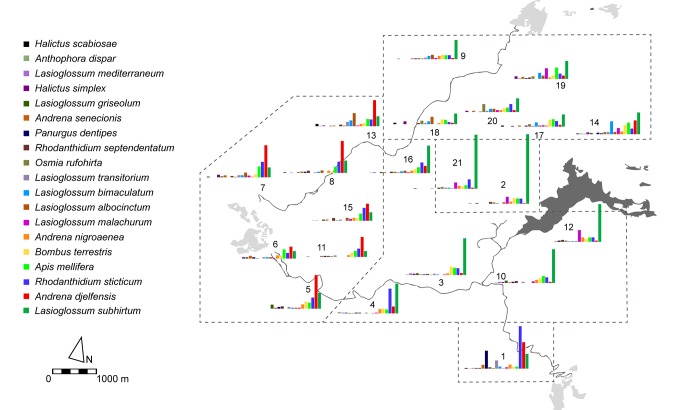
Map of the Garraf Park showing the abundance of the 19 most abundant bee species (representing more than 0.5% of the specimens sampled) in each plot (n = 21). Plots grouped based on bee composition according to cluster analysis.

Neither bee abundance (I = −0.05, p = 0.99) nor species richness (I = 0.002, p = 0.17) showed spatial autocorrelation. Instead, bee composition did show significant autocorrelation (Mantel r = 0.27; p = 0.003). When we analyzed the 19 most abundant species separately, we found spatial autocorrelation for 9 of them (Table 1). Significant autocorrelation occurred mostly at distances <950 m. Importantly, species showing significant autocorrelation had lower body weight (mean ± SD: 20±13 mg; n = 9) than those with no significant autocorrelation (100±72 mg; n = 10) (Table 1; Mann-Whitney U: Z = −2.858; p = 0.004). The cluster analysis of the plots based on bee composition similarity resulted in five groups and revealed a clear geographical pattern (Fig. 3). Interestingly, the two most abundant species, *Lasioglossum subhirtum* and *Andrena djelfensis*, showed partially segregated distributions. *Lasioglossum subhirtum* was dominant in the central and western areas of the park, whereas *A. djelfensis* was dominant on the eastern side. Abundance of these two species showed a significant negative correlation (r_s_ = −0.62; p = 0.003). Other species also showed a geographical pattern. *Lasioglossum malachurum* was most abundant in the NE side, *Lasioglossum bimaculatum* in the N and NW, and *Lasioglossum albocinctum* in the N. *Panurgus dentipes* was abundant only in plot 1, with a bee composition markedly different from that of all other plots. On the other hand, species such as *Rhodanthidium sticticum*, *Apis mellifera*, *Andrena nigroaenea* and *Bombus terrestris* showed a much more homogeneous distribution throughout the park. We calculated the coefficient of variation (n = 21 plots) of the abundance of the 19 main species as a measure of their degree of spatial heterogeneity. Species with higher coefficients of variation (>0.95) had lower body weight (mean ± SD: 27.4±25.8 mg; n = 11) than those with lower (<0.90) coefficients of variation (10.9±76.4 mg; n = 8) (Table 1; Mann-Whitney U: Z = 2.766; p = 0.006), corroborating the conclusion that the observed spatial pattern was mostly due to small species.

**Table 1:** Parameters of the 19 most abundant bee species in the Garraf community.

	Abundance	Moran's I	P	CV of abundance	Body weight (mg)	Nesting substrate	Pollen specialization	Sociality
*Lasioglossum griseolum*	71	0.024	**0.04**	1.12	3.8	Soil	Polylectic	?
*Lasioglossum transitorium*	147	−0.05	0.9	0.99	7.6	Soil	Polylectic	?
*Lasioglossum mediterraneum*	46	0.034	**0.03**	1.24	12.4	Soil	Polylectic?	?
*Lasioglossum malachurum*	222	0.08	**0.001**	1.22	13.3	Soil	Polylectic	Social
*Andrena djelfensis*	926	0.131	**0**	1.03	15.0	Soil	Polylectic?	Solitary
*Lasioglossum subhirtum*	1780	0.045	**0.01**	0.73	16.4	Soil	Polylectic	?
*Panurgus dentipes*	117	−0.026	**0.03**	2.59	18.9	Soil	Oligolectic	Solitary
*Lasioglossum bimaculatum*	202	0.083	**0**	1.21	20.0	Soil	Polylectic?	Solitary
*Osmia rufohirta*	122	0.109	**0**	1.16	27.4	Snail shells	Polylectic	Solitary
*Andrena senecionis*	86	−0.038	0.7	0.89	40.0	Soil	Oligolectic?	Solitary
*Halictus simplex*	71	−0.077	0.5	1.13	40.0	Soil	Polylectic	Solitary?
*Lasioglossum albocinctum*	208	0.034	**0.01**	1.2	49.4	Soil	Polylectic	Solitary
*Rhodanthidium septemdentatum*	121	−0.006	0.3	0.5	85.9	Snail shells	Polylectic	Solitary
*Andrena nigroaenea*	228	−0.024	0.5	0.41	86.5	Soil	Polylectic	Solitary
*Halictus scabiosae*	38	−0.02	0. 3	1.64	93.6	Soil	Polylectic	Social
*Apis mellifera*	528	0.011	0.1	0.37	97.4	Large cavities	Polylectic	Social
*Rhodanthidium sticticum*	817	−0.011	0.2	0.87	103.2	Snail shells	Polylectic	Solitary
*Anthophora dispar*	46	−0.024	0. 5	0.8	188.4	Soil	Polylectic?	Solitary
*Bombus terrestris*	354	−0.015	0.4	0.47	250.5	Large cavities	Polylectic	Social

Abundance (number of specimens captured), Moran's I (significant *p*-values in bold), coefficient of variation of abundance (n = 21 plots), fresh female body weight, of the 19 most abundant species in the Garraf bee community. Species ordered by increasing weight.

### Relationship between Resources and Bee Spatial Distribution

Bee species richness was not related to flower species richness (r^2^ = 0.05; p = 0.32).

However, this lack of relationship was caused by plot 1 (with the highest bee richness and a rather unique bee composition) strongly deviating from the general trend shown by the rest of the plots. Exclusion of this plot would cause the flower-bee richness relationship to become significant (r^2^ = 0.25; p = 0.02). The selected GLM explaining bee richness included no nesting substrate variables, and only one flower variable (*Cistus* flower abundance), but with a non-significant p-value (r^2^ = 0.07, p = 0.122; Table S4). Bee abundance was not related to overall flower abundance (r^2^ = 0.05; p>0.3). The best model explaining bee abundance included abundance of *Cistus* and *T. vulgaris* flowers (r^2^ = 0.32). However, only abundance of *Cistus* flowers was significant (p = 0.013; abundance of *T. vulgaris* flowers, p = 0.169). As with bee richness, bee abundance was not related to nesting substrate availability (Table S4).

**Figure 4:**
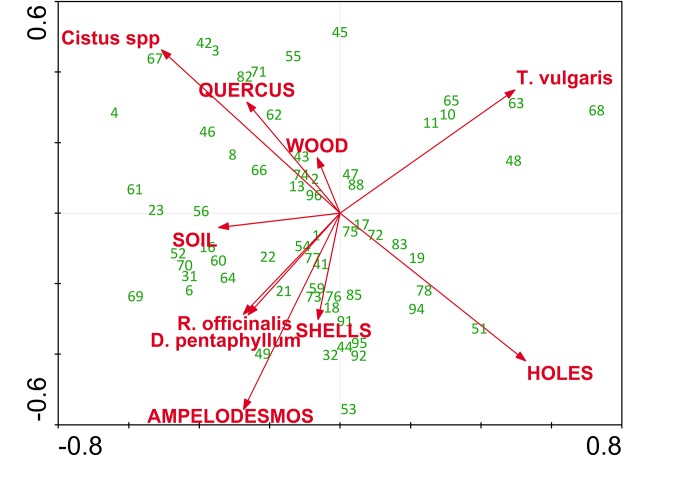
Biplot of RDA model relating small bee species (<55 mg) to flower and nesting resources. Arrows represent resources (flowers in lowercase, nesting substrates in uppercase), and numbers bee species. For species names see Table S1.

The RDA of small species (<55 mg) indicates that the spatial distribution of bee composition is clearly associated to flower resources and only weakly to nesting resources (Fig. 4). Two flower variables were significant in the model: *T. vulgaris* (Contribution to the model = 11.7%; p = 0.01) and *Cistus* spp. (Contribution to the model = 9.8%; p = 0.006). The model including all variables was significant (p = 0.02) and explained 45.2% of the observed variance (Table 2). The first axis explained 25.4% of the variance and was defined by *T. vulgaris* flowers and number of holes in rocks on the one hand, and by *Cistus* spp. flowers on the other hand (Fig. 4). The second axis explained only 5.3% of the variance. On the other hand, the RDA model of large species was non-significant. The overall variance explained was lower (38.9%; Table 2), and no variables entered the model.

**Table 2:** Cumulative variance explained by RDA models relating flower and nesting resources to bee species composition.

	Axis 1	Axis 2	Axis 3	Axis 4	Total Variance
**Small species (n = 62)**					
Cumulative percentage of species variance	38.2	46.3	52.0	56.5	
Cumulative percentage of species-environment variance	56.1	67.8	76.2	82.8	
Sum of canonical eigenvalues					0.452
**Large species (n = 36)**					
Cumulative percentage of species variance	15.3	23.8	31.6	36.8	
Cumulative percentage of species-environment variance	30.7	47.7	63.3	73.8	
Sum of canonical eigenvalues					0.389

## Discussion

The Garraf bee community shows a clear spatial pattern at the habitat scale, with different species dominating in different plots separated by as few as 500–1000 m. This pattern is due to small-sized species (<55 mg), with larger species showing a more or less homogeneous distribution. A likely explanation for this outcome is that our inter-plot distance was sufficient to accommodate the foraging areas of small bees but not those of large species. A positive relationship between body size and foraging areas has been well established [22], [24], [25], [27]. The methods used in these and other related studies, however, tend to provide estimates of either minimum or maximum foraging ranges. Actual foraging distances have been shown to change in time and space based on resource availability [26]–[28], [44]–[48]. Our results provide indirect evidence that, in natural habitats with abundant flower resources, species smaller than 55 mg tend to forage within a radius of 250–500 m. Due to their low food requirements [49], small species may be able to obtain sufficient pollen-nectar resources within a small foraging radius. In parallel studies in our study area we have observed *Lasioglossum transitorium* females (body size: 7.6 mg) completing entire foraging bouts on single *R. officinalis* plants (which may display hundreds of open flowers) located within 50 cm of their nest.

In addition to foraging flights, our plots could be linked by dispersal movements. To our knowledge, information on bee dispersal distances is mostly lacking, but some evidence suggests dispersal distances of at least a few km. Marked *Osmia cornuta* females (a solitary species slightly larger than *Apis mellifera*) have been found nesting 2 km away from their release site [50]. There is also evidence that *Bombus* species may disperse as much as 3–10 km [26]. Even assuming smaller dispersal distances for smaller bees, and given the lack of physical and environmental barriers in the Garraf scrubland, any species should be able to cover the limits of our study area over one or a few generations. Therefore, the fact that our bee community shows such a clear spatial pattern suggests a strong influence of environmental conditions at a very local scale, at least for small species.

Nesting resources show an irregular mosaic distribution across the park. They are not good predictors of bee abundance and richness, and only account for a small part of the explained variance of bee composition. In our community, most species (62%, including 13 of the 19 most abundant) nest underground or are cleptoparasitic on species nesting underground. At the same time, patches of bare soil are abundant and widely distributed across the park, suggesting that they may not be a limiting resource. Species with more specialized nesting habits may be more conditioned by nesting substrate availability. For example, abundance of *O. rufohirta* was marginally associated to abundance of vacant snail shells (r = 0.41, p = 0.06).

Flower resources also show heterogeneity across the park, but in comparison to nesting resources, their distribution shows more of a geographical pattern. Flowers clearly play a greater role than nesting substrates in structuring the spatial distribution of our bee community, accounting for a good part of the explained variance in abundance and composition. This outcome is in agreement with the few studies considering both types of resources [7], [9]. It is important to note that these results should not necessarily be interpreted in terms of evolutionary pollen specialization. For instance, abundance of *L. subhirtum*, the most abundant species, was positively correlated to *T. vulgaris* flower density (r = 0.74, p = 0.0001). However, *L. subhirtum* is clearly polylectic [51], and in Garraf we have observed females of this species (n = 45) foraging on 13 plant species belonging to 7 plant families. Other strong associations involving polylectic species include *Lasioglossum albocinctum* with *D. penthaphyllum* (r = 0.63, p = 0.002) and *L. transitorium* with *Cistus* spp. (r = 0.53, p = 0.01). Oligolectic species make up an important fraction of our bee community (21 oligolectic species, 45 polylectic, 13 cleptoparasitic, and 19 unknown), but only one positively known oligolege, the Asteraceae specialist *Panurgus dentipes*, was among the 19 most abundant species. The remaining oligolectic species were rare, often represented by one or a few individuals, and mostly visiting non-abundant plants in the Asteraceae, Brassicaceae, Ranunculaceae and Boraginaceae. Several studies have found a positive relationship between flower and bee species richness [9], [12], [13], [52], [53]. In Garraf, this relationship was non-significant but, as mentioned, this was caused by a single site (plot 1) displaying a unique bee composition and strongly deviating from the general trend.

Notwithstanding the significant effects of flower resources, as much as 54.8% of the variance in spatial distribution of the Garraf bee community remains unexplained. In addition to resource distribution, community assembly dynamics depend on immigration events and interactions between species. Immigration history (for example, the order of species arrival at a site) may strongly influence the final outcome in terms of species composition [6]. Because our plots are located across an area of contiguous habitat it is fair to assume high levels of dispersal among patches, which would tend to homogenize bee distribution. However, immigration events from outside the habitat [6] and local differences in natural mortality factors such as predation and parasitism, as well as competitive interactions among bee species [7] may contribute to the maintenance of local differences in community composition. We found a negative association between the two most abundant species, *Lasioglossum subhirtum* and *Andrena djelfensis*, whose flight periods overlap widely, but we do not have the necessary information to establish whether this pattern might be attributed to competition. Another factor that could partially explain the geographical pattern observed is phylopatry. The tendency of females to nest at their natal nesting site has been shown in some bee species and could contribute to the increase of local bee density following colonization of a given patch [54], [55]. Other unmeasured environmental factors such as topoclimatic variation could also contribute to the observed bee composition pattern. Daily maximum and minimum temperatures may vary as much as 8°C among microsites distant only few hundred meters from each other [56]. Some studies have found pollinator composition of individual plants to be highly influenced by small-scale variation in microclimatic factors such as solar irradiance, shading and soil wetness [36], [57]. In addition to trying to elucidate the factors responsible for the unexplained spatial variation observed, it would be important to establish whether the observed pattern is stable in time. We do not expect nesting substrate availability to vary much from one year to the next, but blooming intensity is well known fluctuate widely from year to year [58]–[60], potentially affecting bee foraging areas.

Our study demonstrates that bee communities may display clear patterns of spatial heterogeneity at a relatively small scale (500–1000 m) in areas of contiguous suitable habitat and in the absence of local barriers. Importantly, the observed heterogeneity is not irregular, but follows a geographical pattern, and is only partly explained by flower availability. This result is remarkable because bees are highly mobile organisms (both in terms of foraging and dispersal), and therefore one might expect a more homogeneous distribution. Because different bee species have different flower preferences and differ in their pollinating abilities, our results have important implications for local pollination dynamics. Several studies have found differences in reproductive success among populations visited by different pollinators [61]–[63]. Our study suggests that differences in pollination levels may also occur within a plant population as a result of heterogeneous local pollinator distribution. Our results also have important consequences for the study of spatial variation of plant-pollinator networks [36], [64], as overall pollinator community composition may be changing at smaller scales than previously thought.

## Supporting Information

Table S1 **Bee species, their code numbers and body size category.**
(DOC)

Table S2 **Mean and coefficient of variation (n = 21 plots) of flower and nesting resource variables.**
(DOC)

Table S3 **List of flowering plant species.**
(DOC)

Table S4 **Model selection based on Akaike's Information Criterion (AIC).**
(DOC)
