# Supplementary material for: Determinants of Spatial Distribution in a Bee Community: Nesting Resources, Flower Resources, and Body Size
Source: PLoS One. 2014 May 13;9(5):e97255. doi: 10.1371/journal.pone.0097255 (PMC4019551; doi:10.1371/journal.pone.0097255)
Supplement: Table S1 — Bee species, their code numbers and body size category. (DOC) [file pone.0097255.s001.doc]

**Table S1.** Bee species, their code numbers and body size category.

| **Family** | **Species** | **Code number** | **Body size** |
| --- | --- | --- | --- |
| Andrenidae | *Andrena angustior* | 1 | Small |
|  | *Andrena cinerea* | 2 | Small |
|  | *Andrena combinata* | 3 | Small |
|  | *Andrena djelfensis* | 4 | Small |
|  | *Andrena ferrugineicrus* | 5 | Large |
|  | *Andrena fertoni* | 6 | Small |
|  | *Andrena flavipes* | 7 | Large |
|  | *Andrena hesperia* | 8 | Small |
|  | *Andrena labialis* | 9 | Large |
|  | *Andrena lagopus* | 10 | Small |
|  | *Andrena lepida* | 11 | Small |
|  | *Andrena limata* | 12 | Large |
|  | *Andrena livens* | 13 | Small |
|  | *Andrena nigroaenea* | 14 | Large |
|  | *Andrena nigroolivacea* | 15 | Large |
|  | *Andrena senecionis* | 16 | Small |
|  | *Andrena similis* | 17 | Small |
|  | *Andrena solenopalpa* | 18 | Small |
|  | *Andrena sp.* | 19 | Small |
|  | *Andrena trimmerana* | 20 | Large |
|  | *Andrena verticalis* | 21 | Small |
|  | *Andrena vulpecula* | 22 | Small |
|  | *Panurgus dentipes* | 23 | Small |
| Apidae | *Amegilla quadrifasciata* | 24 | Large |
|  | *Anthophora acervorum* | 25 | Large |
|  | *Anthophora dispar* | 26 | Large |
|  | *Apis mellifera* | 27 | Large |
|  | *Bombus pascuorum* | 28 | Large |
|  | *Bombus pratorum* | 29 | Large |
|  | *Bombus terrestris* | 30 | Large |
|  | *Ceratina cucurbitina* | 31 | Small |
|  | *Ceratina cyanea* | 32 | Small |
|  | *Eucera alternans* | 33 | Large |
|  | *Eucera caspica* | 34 | Large |
|  | *Eucera chrysopyga* | 35 | Large |
|  | *Eucera collaris* | 36 | Large |
|  | *Eucera elongatula* | 37 | Large |
|  | *Eucera nigrilabris* | 38 | Large |
|  | *Eucera taurica* | 39 | Large |
|  | *Melecta luctuosa* | 40 | Large |
|  | *Nomada connectens* | 41 | Small |
|  | *Nomada dicrepans* | 42 | Small |
|  | *Nomada discedens* | 43 | Small |
|  | *Nomada flavoguttata* | 44 | Small |
|  | *Nomada hispanica* | 45 | Small |
|  | *Nomada integra* | 46 | Small |
|  | *Nomada panurgina* | 47 | Small |
|  | *Nomada serricornis* | 48 | Small |
|  | *Nomada sheppardana* | 49 | Small |
|  | *Xylocopa violacea* | 50 | Large |
| Colletidae | *Hylaeus garrulus* | 51 | Small |
|  | *Hylaeus gibbus* | 52 | Small |
|  | *Hylaeus hyalinatus* | 53 | Small |
|  | *Hylaeus taeniolatus* | 54 | Small |
| Halictidae | *Halictus fulvipes* | 55 | Small |
|  | *Halictus gemmeus* | 56 | Small |
|  | *Halictus quadricinctus* | 57 | Large |
|  | *Halictus scabiosae* | 58 | Large |
|  | *Halictus simplex* | 59 | Small |
|  | *Lasioglossum albocinctum* | 60 | Small |
|  | *Lasioglossum bimaculatum* | 61 | Small |
|  | *Lasioglossum griseolum* | 62 | Small |
|  | *Lasioglossum ibericum* | 63 | Small |
|  | *Lasioglossum interruptum* | 64 | Small |
|  | *Lasioglossum malachurum* | 65 | Small |
|  | *Lasioglossum mediterraneum* | 66 | Small |
|  | *Lasioglossum morio* | 67 | Small |
|  | *Lasioglossum subhirtum* | 68 | Small |
|  | *Lasioglossum transitorium* | 69 | Small |
|  | *Sphecodes pseudofasciatus* | 70 | Small |
|  | *Sphecodes puncticeps* | 71 | Small |
|  | *Sphecodes ruficrus* | 72 | Small |
| Megachilidae | *Chelostoma florisomne* | 73 | Small |
|  | *Hoplitis (Anthocopa) sp.* | 74 | Small |
|  | *Hoplitis adunca* | 75 | Small |
|  | *Hoplitis anthocopoides* | 76 | Small |
|  | *Hoplitis benoisti* | 77 | Small |
|  | *Hoplosmia ligurica* | 78 | Small |
|  | *Megachile baetica* | 79 | Large |
|  | *Megachile ericetorum* | 80 | Large |
|  | *Megachile pyrenaica* | 81 | Large |
|  | *Osmia aurulenta* | 82 | Small |
|  | *Osmia gallarum* | 83 | Small |
|  | *Osmia latreillei* | 84 | Large |
|  | *Osmia melanogaster/leaiana* | 85 | Small |
|  | *Osmia mustelina* | 86 | Large |
|  | *Osmia nasoproducta* | 87 | Large |
|  | *Osmia nasuta* | 88 | Small |
|  | *Osmia niveata* | 89 | Large |
|  | *Osmia niveocincta* | 90 | Large |
|  | *Osmia rufohirta* | 91 | Small |
|  | *Osmia submicans* | 92 | Small |
|  | *Osmia tricornis* | 93 | Large |
|  | *Protosmia (Nanosmia) sp.* | 94 | Small |
|  | *Protosmia capitata* | 95 | Small |
|  | *Protosmia exenterata* | 96 | Small |
|  | *Rhodanthidium septemdentatum* | 97 | Large |
|  | *Rhodanthidium sticticum* | 98 | Large |
