# Supplementary material for: Determinants of Spatial Distribution in a Bee Community: Nesting Resources, Flower Resources, and Body Size
Source: PLoS One. 2014 May 13;9(5):e97255. doi: 10.1371/journal.pone.0097255 (PMC4019551; doi:10.1371/journal.pone.0097255)
Supplement: Table S2 — Mean and coefficient of variation (n = 21 plots) of flower and nesting resource variables. (DOC) [file pone.0097255.s002.doc]

**Table S2. Mean and coefficient of variation (n= 21 plots) of flower and nesting resource variables.**

|  | **Mean** | **CV** |
| --- | --- | --- |
| **Flowers** |  |  |
| Flower abundance (flowers / m^2^) | 110.58 | 0.61 |
| Flower richness | 15.57 | 0.34 |
| *Dorycnium pentaphyllum* flowers / m^2^ | 8.87 | 1.15 |
| *Rosmarinus officinalis* flowers / m^2^ | 52.01 | 0.74 |
| *Thymus vulgaris* flowers / m^2^ | 49.11 | 1.03 |
| *Cistus* spp. flowers / m^2^ | 0.59 | 1.54 |
| **Nesting resources** |  |  |
| % Bare soil | 13.1 | 0.71 |
| % Dead wood | 0.1 | 2.54 |
| % *Quercus coccifera* cover | 12.9 | 1.08 |
| % *Ampelodesmos mauritanica* cover | 6.1 | 1.08 |
| Number of holes in rocks / m^2^ | 0.55 | 1.28 |
| Number of snail shells / m^2^ | 0.19 | 1.40 |
