# Supplementary material for: Determinants of Spatial Distribution in a Bee Community: Nesting Resources, Flower Resources, and Body Size
Source: PLoS One. 2014 May 13;9(5):e97255. doi: 10.1371/journal.pone.0097255 (PMC4019551; doi:10.1371/journal.pone.0097255)
Supplement: Table S3 — List of flowering plant species. (DOC) [file pone.0097255.s003.doc]

**Table S3.** List of flowering plant species.

| **Flower species** |
| --- |
| *Allium roseum* |
| *Allium sphaerocephalon* |
| *Anagallis arvensis* |
| *Antirrhinum barrelieri* |
| *Aphyllanthes monspeliensis* |
| *Argyrolobium zanonii* |
| *Aristolochia pistolochia* |
| *Asperula cynanchia* |
| *Biscutella laevigata* |
| *Centaurea linifolia* |
| *Centaurea montana ssp. semidecurrens* |
| *Centaurium erythraea* |
| *Cistus albidus* |
| *Cistus monspeliensis* |
| *Cistus salviifolius* |
| *Clematis flammula* |
| *Convolvulus lanuginosus* |
| *Coris monspeliensis* |
| *Cytisophyllum sessilifolium* |
| *Dorycnium hirsutum* |
| *Dorycnium pentaphyllum* |
| *Echium vulgare* |
| *Erica multiflora* |
| *Euphorbia characias* |
| *Euphorbia flavicoma* |
| *Fumana ericifolia* |
| *Fumana ericoides* |
| *Fumana laevipes* |
| *Fumana laevis* |
| *Galium aparine* |
| *Galium palustre* |
| *Genista scorpius* |
| *Gladiolus illyricus* |
| *Helianthemum oelandicum spp.italicum* |
| *Helichrysum stoechas* |
| *Hippocrepis comosa* |
| *Hypericum perforatum* |
| *Lathyrus* |
| *Leuzea conifera* |
| *Linum strictum* |
| *Lithospermum fruticosum* |
| *Lonicera implexa* |
| *Muscari neglectum* |
| *Narcissus assoanus* |
| *Ononis minutissima* |
| *Orobanche latisquama* |
| *Phlomis lychnitis* |
| *Polygala rupestris* |
| *Potentilla sp.* |
| *Psoralera bituminosa* |
| *Ranunculus gramineus* |
| *Rosmarinus officinalis* |
| *Rubia peregrina* |
| *Scorpiurus muricatus* |
| *Sedum sediforme* |
| *Sideritis hirsuta* |
| *Teucrium chamaedrys* |
| *Thalictrum tuberosum* |
| *Thesium divaricatum* |
| *Thymus vulgaris* |
| *Torilis arvensis* |
| *Vicentoxicum hirundinaria* |
| *Vicentoxicum nigrum* |
| *Vicia cracca* |
