# Supplementary material for: Determinants of Spatial Distribution in a Bee Community: Nesting Resources, Flower Resources, and Body Size
Source: PLoS One. 2014 May 13;9(5):e97255. doi: 10.1371/journal.pone.0097255 (PMC4019551; doi:10.1371/journal.pone.0097255)
Supplement: Table S4 — Model selection based on Akaike's Information Criterion (AIC). (DOC) [file pone.0097255.s004.doc]

**Table S4.** Model selection based on Akaike’s Information Criterion (AIC).

| Dependent variable | Independent variables | | | | | | | | | k | AIC | ΔAIC |
| --- | --- | --- | --- | --- | --- | --- | --- | --- | --- | --- | --- | --- |
|  | Intercept | Roff | Tvul | Dpen | Cis | Dw | Bs | Hinr | snail |  |  |  |
| Species richness | x | x | x | x | x | x | x | x | x | 9 | 137.84 | 10.45 |
|  | x | x | x | x | x | x |  | x | x | 8 | 136.14 | 8.75 |
|  | x | x | x |  | x | x |  | x | x | 7 | 134.46 | 7.07 |
|  | x | x | x |  | x |  |  | x | x | 6 | 133.29 | 5.9 |
|  | x |  | x |  | x |  |  | x | x | 5 | 132.07 | 4.68 |
|  | x |  | x |  | x |  |  | x |  | 4 | 130.52 | 3.13 |
|  | x |  |  |  | x |  |  | x |  | 3 | 129.07 | 1.68 |
|  | **x** |  |  |  | **x** |  |  |  |  | **2** | **127.39** | **0.00** |
|  | x |  |  |  |  |  |  |  |  | 1 | 128.10 | 0.71 |
| Abundance | x | x | x | x | x | x | x | x | x | 9 | 11.37 | 10.91 |
|  | x | x | x | x | x |  | x | x | x | 8 | 9.38 | 8.92 |
|  | x |  | x | x | x |  | x | x | x | 7 | 7.41 | 6.95 |
|  | x |  | x | x | x |  | x |  | x | 6 | 5.46 | 5.00 |
|  | x |  | x | x | x |  |  |  | x | 5 | 3.64 | 3.18 |
|  | x |  | x | x | x |  |  |  |  | 4 | 1.76 | 1.30 |
|  | **x** |  | **x** |  | **x** |  |  |  |  | **3** | **0.46** | **0.00** |
|  | x |  |  |  | x |  |  |  |  | 2 | 0.73 | 0.27 |
|  | x |  |  |  |  |  |  |  |  | 1 | 5.94 | 5.48 |

Analyses of the relationship of bee species richness and abundance (n=21 plots) with flower and nesting resource variables. Variables included in each model are marked with an x. The selected model is in bold, (k) is the number of parameters in the model and (ΔAIC) is the difference in AIC between the selected model and the given model. Independent variables are density of *R. officinalis* flowers –Roff-; *T. vulgaris* flowers*–*Tvul*-*, *D. pentaphyllum* flowers*–*Dpen*-* , *Cistus* spp. flowers –Cis-, presence of dead wood – Dw-, % of bare soil –Bs-, number of holes in rocks – Hinr- and number of vacant snail shells – snail-.
